# Impact of Ultrasonication on the Self-Assembly Behavior and Gel Properties of Bovine Bone Collagen I

**DOI:** 10.3390/molecules28073096

**Published:** 2023-03-30

**Authors:** Hong Liu, Hongru Zhang, Kangyu Wang, Liwei Qi, Yujie Guo, Chunhui Zhang, Yang Xu

**Affiliations:** 1 Comprehensive Key Laboratory of Agro-Products Processing, Ministry of Agriculture and Rural Affairs, Institute of Food Science and Technology, Chinese Academy of Agricultural Sciences, Beijing 100193, China; 2 Laboratory of Biomass and Green Technologies, University of Liege-Gembloux Agro-Bio Tech, Passage des Déportés 2, B-5030 Gembloux, Belgium; 3 Inner Mongolia Mengtai Biological Engineering Co., Ltd., Hohhot 010000, China

**Keywords:** ultrasonic treatment, collagen fibril, self-assembly behavior, collagen fibril gel, gel property

## Abstract

This study deliberated the effect of ultrasonic treatment on collagen self-assembly behavior and collagen fibril gel properties. Bovine bone collagen I which had undergone ultrasonic treatment with different power (0–400 W) and duration (0–60 min) was analyzed. SDS-PAGE and spectroscopic analysis revealed that ultrasonic treatment decreased collagen molecular order degree and the number of hydrogen bonds, stretching collagen telopeptide regions while maintaining the integrity of the collagen triple-helical structure. Ultrasonic treatment (*p* ≤ 200 W, t ≤ 15 min) dispersed the collagen aggregates more evenly, and accelerated collagen self-assembly rate with a decreased but more homogeneous fibril diameter (82.78 ± 16.47–115.52 ± 19.51 nm) and D-periodicity lengths (62.1 ± 2.9–66.5 ± 1.8 nm) than that of the untreated collagen (119.15 ± 27.89 nm; 66.5 ± 1.8 nm). Meanwhile, ultrasonic treatment (*p* ≤ 200 W, t ≤ 15 min) decreased the viscoelasticity index and gel strength, enhancing thermal stability and promoting specific surface area and porosity of collagen fibril gels than that of the untreated collagen fibril gel. These results testified that collagen self-assembly behavior and collagen fibril gel properties can be regulated by ultrasonic treatment through multi-hierarchical structural alteration. This study provided a new approach for controlling in vitro collagen fibrillogenesis process so as to manufacture novel desirable collagen-based biomaterials with propitious performances for further valorization.

## 1. Introduction

Collagen is the predominant structural protein of extracellular matrix (ECM), constituting approximately 1/3 of the total body proteins [1]. Type I collagen (CI) is the most abundant amongst the identified 29 collagen types (type I–XXIX), and also possess superior biodegradability and biocompatibility, which has now been widely utilized in food, cosmetics, biomedical and pharmaceutical industries [2,3]. Whether in vivo or in vitro under physiological conditions (specific pH, temperature, ionic strength, concentration, etc.), CI monomers can spontaneously aggregate side-by-side with each other in a quarter-staggered manner to self-assemble into microfibrils, fibrils, further into fibril bundles and, eventually, 3D collagen fibril gels [4]. Collagen fibrils and collagen fibril gels organize differently under different conditions through hydrogen bonding and hydrophobic–electrostatic interactions, and maintain optimized physicochemical properties and biological activity in vitro that are similar to those of native tissues [5]. As the most vital strength and viscoelasticity-related protein, the self-assembly behavior and fibril gel properties of CI make it responsible for the structural stability, mechanical properties, and application potentiality of collagen-based products in tissue engineering and traditional meat processing [6,7,8]. Therefore, different collagen self-assembly behavior and gel properties determine the different applied potency of collagen.

Many studies have found that collagen self-assembly behavior and fibril gel properties are strongly affected by relevant assembly conditions, such as collagen type/concentration, temperature, pH, ionic strength/species, surfactants, fibrillation inhibitors, external force field, etc. [4]. As an efficient and economic non-thermal assistant modification approach, low frequencies ultrasound (20–100 kHz, 1–1000 W/cm^2^) has progressively been employed by food sectors as an alternative to conventional processes [9]. During ultrasonic treatment, electrical energy is converted into mechanical vibration energy, producing an acoustic cavitation effect and creating local high temperature and pressure on materials in a liquid system [10]. The acoustic cavitation generates sufficiently strong energy (10–100 kJ/mol) and physical shear forces that can break covalent and non-covalent bonds (e.g., hydrogen bonds and hydrophobic interactions) and ameliorating the physicochemical properties of proteins [11]. Li et al. (2018) reported that the D-spacing length and roughness of collagen fibrils from Qinchuan beef cattle tendons observed a 1.02% increment induced by ultrasonic treatment for 20 min (20, 28 and 40  kHz) using atomic force microscopy [12]. Yu et al. (2020) found that the ultrasonic cavitation and mechanical effect could accelerate the self-assembly process, reduce the fibrils size and alter digestion characteristics of pepsin-soluble collagen extracted from chicken leg skin [13]. Wan et al. (2021) studied the ultrastructure and mechanical properties of ultrasonic treatment collagen fibrils by atomic force microscopy [6].

In our previous study, ultrasonic treatment was applied to the chicken breast cartilage, and the result evinced that ultrasonication is an effective way to increase the yield of type II collagen and improve its physicochemical characteristics [8,9]. However, the potential effect of ultrasonication on the structural integrity, self-assembly behavior of the extracted collagen and the properties of the resulting collagen fibril gels has not yet been well-described, especially for bone collagen I. We have thus particularly recovered and characterized the attributes of bovine bone collagen I to valorize the low-value bovine bone byproducts as a high-quality alternative collagen source [14]. Bearing these in mind, this study aimed to further investigate the effect of ultrasonic treatment (power and time) on the self-assembly behavior and fibril gel properties of the extracted bovine bone collagen I. We expect that valuable data would be furnished for controlling the collagen fibril-formation process in vitro, and would lay a foundation for the fabrication of novel desirable bone collagen-based biomaterials and ECM analogues by ultrasonication.

## 2. Results and Discussion

### 2.1. Collagen Self-Assembly Behavior

#### 2.1.1. Turbidity Assay

**Figure 1:**
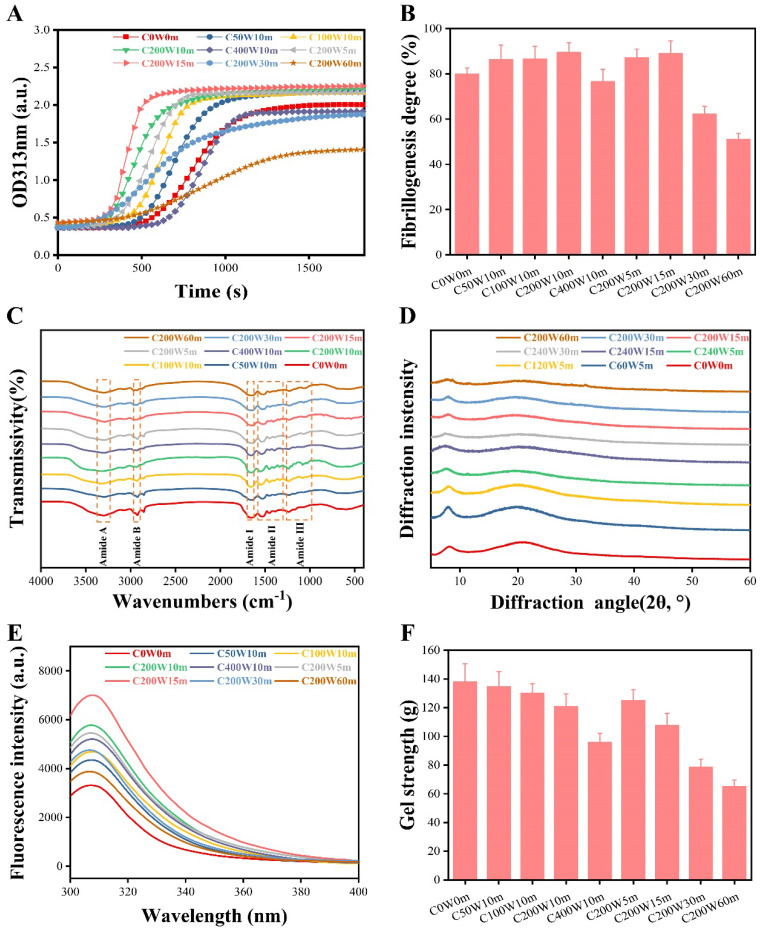
Turbidity assay (**A**), fibrillogenesis degree (**B**), fourier transform infrared spectroscopy (**C**), X-ray diffraction spectra (**D**) and fluorescence emission spectra (**E**) of collagen; gel strength of collagen fibril gels (**F**).

Collagen molecules possess all the information needed for fibrillogenesis so that it could self-assemble to be high-ordered fibrils when exposed to physiological conditions [2]. The collagen turbidimetric curve (Figure 1A) generally comprised three distinct stages to be a sigmoidal profile: lag phase (nucleation stage), growth phase (assembly stage) and plateau phase (equilibrium stage), indicating that the kinetics of collagen fibrillation followed a nucleation-dependent polymerization mechanism [15]. Nucleation occurred by the aggregation of collagen molecules to form dimers during the lag phase without detectable turbidimetric change, and the further aggregation of collagen molecules resulted in the formation of collagen fibrils with a steep sigmoidal increase in turbidity values in the growth phase [16].

Under different ultrasonic treatment power (10 min), the turbidimetric curve of C0W0m exhibited a prolonged lag phase 0 s~590 s (590 s) and a growth phase of 590 s~1200 s (610 s). The turbidimetric curves of C50W10m (0 s~490 s, 490 s; 490 s~1015 s, 525 s), C100W10m (0 s~455 s, 455 s; 455 s~840 s, 385 s) and C200W10m (0 s~315 s, 315 s; 315 s~735 s, 420 s) displayed a shorter nucleation and assembly stage, but not for C400W10m (0 s~665 s, 665 s; 665 s~1085 s, 420 s) when compared to that of C0W0m. Results of the turbidity assay indicated that 50–200 W of ultrasonic treatment gradually increased the collagen fibrillogenesis rate, but 400 W decreased it. The fibrillogenesis degree (Figure 1B) also increased within 0–200 W (80.03–89.53%, 10 min) but decreased significantly at C400W10m (76.68%). These results showed that moderate ultrasonic treatment power accelerated collagen fibrillogenesis process and increased fibrillogenesis degree, with the optimal effect at 200 W. Under different ultrasonic treatment time (0–60 min, 200 W), turbidimetric curves of C200W5m (0 s~385 s, 385 s; 385 s~770 s, 385 s) and C200W15m (0 s~280 s, 280 s; 280 s~595 s, 315 s) showed a gradually increased lag phase and growth phase. However, the turbidimetric curves of C200W30m and C200W60m emerged with an unobvious sigmoidal profile and prolonged lag and growth phase, as well as a decreased equilibrium turbidity than that of C0W0m. In the plateau stage, collagen fibrils further assembled into supramolecular organization accompanied with an equilibrium of turbidity value [17].

These results suggested that moderate ultrasonic treatment (*p* ≤ 200 W, t ≤ 15 min) collagen dispersed the collagen monomer homogeneously and increased the probability of collision and assembly, which could accelerate the nucleation stage so as to increase the fibrillogenesis rate [16]. However, high power and longtime ultrasonic treatment (*p* > 200 W, t > 15 min) weakened the collagen self-assembly ability, possibly because a negative impact was exerted on the structural integrity of collagen induced by cavitation effect [18].

#### 2.1.2. Fibrillogenesis Degree

Fibrillogenesis is an entropy-driven process through hydrophobic and electrostatic interactions between the non-polar regions of adjacent molecules and hydrogen bonding between polar residues [19], as well as the specificity of molecular recognition [20]. These interactive forces minimize the surface area/volume ratio to be a circular cross-section by the loss of solvent molecules from collagen surface [21]. Collagen fibrillogenesis degree (Figure 1b) increased gradually within 0–200 W (80.03–89.53%, 10 min) and 5–15 min (87.26–89.03%, 200 W) but decreased significantly at C400W10m (76.68%), C200W30m (62.26%) and C200W60m (51.18%). These results suggested that moderate ultrasonic treatment (*p* ≤ 200 W, t ≤ 15 min) was conducive to the fibrillogenesis process, but high power and longtime ultrasonic treatment (*p* > 200 W, t > 15 min) impaired it. Ultrasonication had possibly affected the molecular stretching and self-assembly process of collagen, thereby affecting the collagen fibrillogenesis degree [18,22]. The three-stage collagen fibrillogenesis process are generally accepted, yet the self-assembly mechanism is still lesser known [21].

### 2.2. Microstructure of Ultrasonic Treatment Collagen

#### 2.2.1. SDS-PAGE

**Figure 2:**
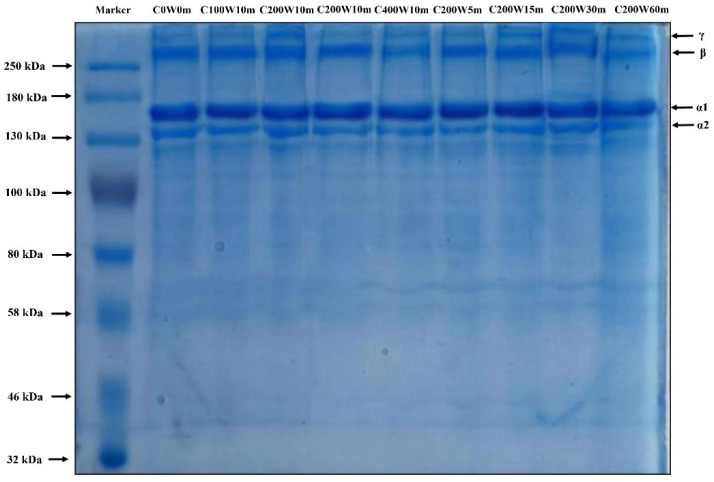
SDS-PAGE electrophoretic pattern of collagen.

SDS-PAGE electrophoretic pattern (Figure 2) showed that all the collagen comprised of α1-, α2-, β- (dimers) and γ-chains (trimers), and the band intensity of α1-chains were approximately two-fold that of α2-chains (Table S2). These results testified that ultrasonic treatment did not destroy the covalent bond of collagen, and the structural integrity of its subunits (α1 and α2 chains) were maintained. The existence of high molecular weight bands (β- and γ-components) suggested that there existed various intra- and/or inter-molecular cross-linkages, which was conducive to maintain the thermal stability of collagen [11]. However, the diminished band intensity of β- and γ-chains was observed in C400W10m, C200W30m and C200W60m instead of C0W0m (Figure 2). These results suggested that high power and longtime ultrasonic treatment (*p* > 200 W, t > 15 min) had possibly induced an adverse impact on the structural and/or thermal stability of collagen.

#### 2.2.2. Fourier Transform Infrared Spectroscopy (FTIR)

The FTIR spectra (Figure 1C) exhibited the collagen distinctive transmissivity peaks of amide A (3440–3400 cm^−1^), B (2980–2850 cm^−1^), I (1700–1600 cm^−1^), II (1600–1550 cm^−1^) and III (1360–1200 cm^−1^) bands (Table S3), suggesting that all the collagen retained the triple-helical structure. The amide A and B bands are associated with free N-H stretching and asymmetric stretch vibrations of C-H and -NH_3_^+^, respectively [14]. The amide I band is a sensitive marker of secondary structure related to the stretching vibration of C=O groups coupled with N–H groups, but will shift to lower wavenumbers for the increased hydrogen bonds generated by the C=O groups with adjacent chains by stretching vibrations [8]. The amide II band is aroused by the N-H bending and CN stretching vibrations, while the amide III band is a weak absorption related to the N-H bending, C-N stretching, N-H bending vibrations and the CH_2_ group wagging vibration in the glycine backbone proline side chains [23,24].

It was found that all the amide A band shifted to approximately 3300 cm^−1^, indicating that the N-H groups were involved in hydrogen bonding with functional groups [25]. With the increase in ultrasonic treatment power and time, the amide B bands transfer from 2294 cm^−1^ to 2932 cm^−1^ due to the higher exposure of N-terminal free NH_3_^+^ groups of lysine residues induced by ultrasonic treatment [8,24]. The amide I band wavenumbers of collagen generally increased with the increased ultrasonic treatment power (1661–1651 cm^−1^) and treatment time (1651–1634 cm^−1^) than that of C0W0m (1653 cm^−1^), proving that ultrasonic treatment decreased the molecular order degree and the number of hydrogen bonds of collagen. The amide II and III band wavenumbers of the ultrasonic treatment collagen (1520–1553 cm^−1^ and 1230–1240 cm^−1^, respectively) were found higher than control (1518 cm^−1^ and 1230 cm^−1^, respectively). These results suggested that non-ultrasonic treatment collagen had higher molecular order and more hydrogen bonds between adjacent α-chains than that of ultrasonic treatment collagen.

Curve-fitting analysis of amide I band of collagen (Table S4) also revealed that C0W0m had a higher content of α-helices + β-sheets (93.27%) than that of ultrasonic treatment collagen (56.96–86.85%), manifesting that ultrasonic treatment may exert adverse effects on the structural integrity of collagen. Furthermore, the peak intensity ratio (A_Ⅲ_/A_1 450_) represents a well-maintained and intact triple-helical structure if it is 1.05–1.14 [14]. A_Ⅲ_/A_1 450_ of all the ultrasonic/non-ultrasonic treatment collagen was 1.0487–1.1299 (Table S3), further proving that all the collagen maintained intact triple-helical structure. However, high power and longtime ultrasonic treatment (*p* > 200 W, t > 15 min) may weaken the structural integrity of collagen and induced multi-hierarchical structural differences, such as the number of hydrogen bonds, degree of molecular order, etc. These multi-hierarchical structural differences induced by ultrasonic treatment might greatly influence the physicochemical properties or biological activities of collagen.

#### 2.2.3. X-ray Diffraction Spectra (XRD)

Two diffraction peaks at diffraction angles (2θ) aroused from the triple-helical structure and diffuse scatter caused by many structural layers of collagen, respectively, were observed in XRD spectra (Figure 1D). The result of the XRD spectra suggested that all the collagen retained their native triple-helical conformations and crystallinity [26]. The d values were calculated by the Bragg equation and represented the distance between crystal planes (Table S5). The d values of the first sharp peak (d1) and the second broad peak (d2) of collagen reflected the distance between the molecular chains and the distance between the skeletons of collagen, respectively [27]. The d1 values (related to the diameter of the triple-helix structure) of ultrasonic treatment collagen (1.107–1.174 nm) increased with the increased ultrasonic treatment power (50–400 W) and time (5–60 min) than that of C0W0m (1.088 nm). While the d2 values (related to the distance between amino acid residues along the helix of collagen) of ultrasonic treatment collagen (0.426–0.452 nm) increased within 50–200W (10 min) and 5–30 min (200W), they decreased at 400 W (10 min) and 60 min (200W) compared to that of C0W0m (0.425 nm). Consequently, the results of XRD showed that ultrasonic treatment did not damage the structural integrity but expanded the mainly intermolecular and then intramolecular distances of collagen molecules. The greater distance between the molecular chains of ultrasonic treatment collagen, such as C400W10m, C200W30m, and C200W60m, may make it more suitable as a drug delivery carrier than C0W0m [18].

#### 2.2.4. Fluorescence Emission Spectra

The maximum fluorescence intensity of collagen fibrils (Figure 1E) increased with the increase of ultrasonic treatment power from 0 to 200 W, but decreased at 400 W (10 min). The results suggested that that the cavitation effect of ultrasonication induced the extension of the collagen molecules and fibrils, thereby exposing the inter- and intra-molecular tyrosine residues and increasing the fluorescence intensity. The maximum fluorescence intensity also increased with the extension of ultrasonic treatment time (5, 15 min) but decreased at 30 and 60 min (200 W). Tyrosine (intrinsic fluorophore) was present only at the N- and C-terminal telopeptide regions of collagen molecules and was responsible for initiating collagen fibrillation [28]. Therefore, it is speculated that the moderate ultrasonic cavitation effect reinforced the molecular stretching degree mainly through the exposure of tyrosine residues in the telopeptide regions of collagen molecules, thereby increasing the fluorescence intensity. However, high power and longtime ultrasonic treatment (*p* > 200 W, t > 15 min) may induce a smaller number of collagen self-assembled aggregates with a greater aggregation degree, thus reducing the fluorescence intensity.

#### 2.2.5. Collagen Fibril Morphology (SEM/TEM)

**Figure 3:**
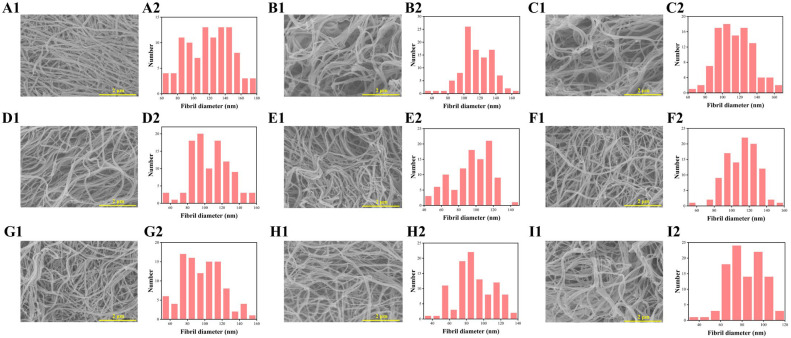
SEM images of collagen fibrils of C0W0m (**A1**), C50W10m (**B1**), C100W10m (**C1**), C200W10m (**D1**), C400W10m (**E1**), C200W5m (**F1**), C200W15m (**G1**), C200W30m (**H1**), C200W60m (**I1**). The fibril-diameter distribution of C0W0m (**A2**), C50W10m (**B2**), C100W10m (**C2**), C200W10m (**D2**), C400W10m (**E2**), C200W5m (**F2**), C200W15m (**G2**), C200W30m (**H2**) and C200W60m (**I2**).

**Figure 4:**
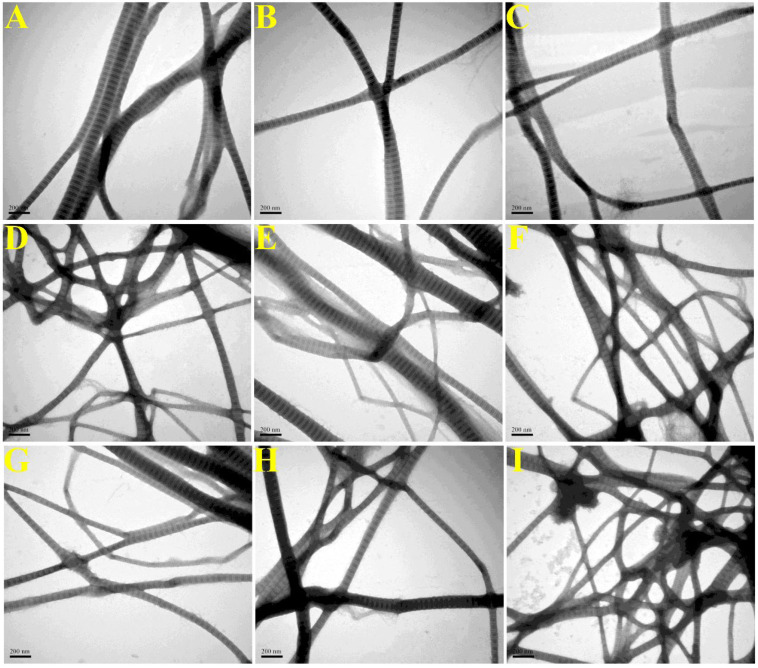
TEM images of collagen fibrils of C0W0m (**A**), C50W10m (**B**), C100W10m (**C**), C200W10m (**D**), C400W10m (**E**), C200W5m (**F**), C200W15m (**G**), C200W30m (**H**) and C200W60m (**I**).

SEM/TEM was adopted to observe the morphology of collagen fibrils. As was shown in Figure 3A1–I1, collagen fibrils were entangled into a dense and delicate fibrillar network with porous structure observed by SEM, possibly due to dehydration by freeze drying [18]. The average fibril diameters (Figure 3A2–I2) of C0W0m (119.15 ± 27.89 nm), C50W10m (115.52 ± 19.51 nm), C100W10m (113.63 ± 20.53 nm), C200W10m (105.35 ± 21.82 nm) and C400W10m (93.86 ± 22.05 nm) decreased gradually, and the average fibrils diameters of C200W5m (110.56±17.58 nm), C200W15m (96.33 ± 23.25 nm), C200W30m (88.38 ± 22.03 nm) and C200W60m (82.78 ± 16.47 nm) also exhibited a decreased trend. As for the characteristic collagen fibrils D-periodicity structure (Figure 4A–I), the average D-periodicity lengths of C0W0m (66.5 ± 1.8 nm), C50W10m (65.8 ± 2.2 nm), C100W10m (64.9 ± 2.6 nm), C200W10m (63.5 ± 1.6 nm) and C400W10m (62.1 ± 2.9 nm) decreased with the increase of ultrasonic treatment power (0-400 W), and the average D-periodicity lengths of C200W5m (64.1 ± 2.0 nm), C200W15m (62.8 ± 2.3 nm), C200W30m (61.7 ± 3.2 nm) and C200W60m (62.1 ± 3.6 nm) also exhibited a decreased trend.

Results of SEM/TEM demonstrated that ultrasonic treatment did not interrupt collagen fibrillogenesis and formation of D-periodicity, but diminished the fibril diameter and D-periodicity length of ultrasonic treatment collagen when compared with C0W0m. Furthermore, the fibril-diameter distribution of ultrasonic treatment collagen was more homogeneous. Previous studies had concluded that ultrasonic treatment mainly accelerate the collagen self-assembly process at the nucleation stage through increasing collagen molecular interaction [7]. Meanwhile, ultrasonic treatment dispersed trivalent phosphate ions in solution evenly through the cavitation effect to widely bind on the high excess positively charged regions of collagen molecules to accelerate the collagen self-assembly process [7]. However, collagen fibrillogenesis became a spontaneous and orderly molecular arrangement process after the formation of collagen fibril nucleus, and thereby ultrasonic treatment has no significant effect on the kinetics of self-assembly and the equilibrium stage.

The fibril diameters and D-periodicity width of ultrasonic treatment collagen exhibited lower heterogeneity than that of control (C0W0m). In this regard, the collagen monomer molecules accumulated randomly into microfibrils at the nucleation stage, and then the microfibrils further assembled into fibrils, resulting in the heterogeneous distribution of microfibril and fibril diameters [13]. Ultrasonic treatment may disperse the collagen monomers more evenly through cavitation effect, increasing the number of collagen microfibrils formed in the nucleation stage. Therefore, the increased number of collagen microfibrils will decrease the average fibril diameter and fibril diameter heterogeneity when the number of total collagen molecules is constant. Mechanisms of D-periodicity formation are still not well-understood, but ultrasonic treatment may affect the length of gap areas and overlapping areas through variable molecular arrangement, resulting in the smaller D-periodicities according to the 1/4 staggered model of Schmitt hypothesis [19,29]. The collagen fibrils with smaller diameter interact more affine with ECM components, facilitating elasticity and decreased plastic deformation [2]. The uniform morphology of collagen fibrils (diameter size distributions, network structure, etc.) was a significant favorable property for biomedical and pharmaceutical applications [30].

### 2.3. Thermal Stability of Collagen Fibril Gels

#### 2.3.1. Differential Scanning Calorimetry (DSC)

**Figure 5:**
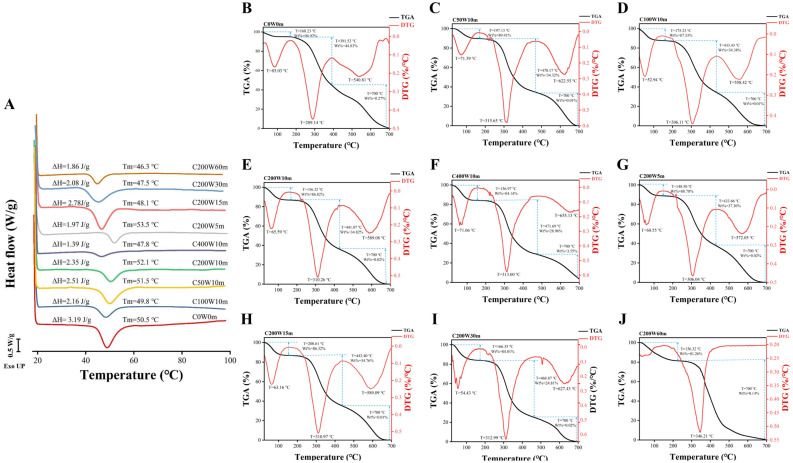
Differential scanning calorimetry curves of collagen fibril gels at 20–100 °C (**A**); thermogravimetric curves (TGA) and its first derivatives (DTG) of C0W0m (**B**), C50W10m (**C**), C100W10m (**D**), C200W10m (**E**), C400W10m (**F**), C200W5m (**G**), C200W15m (**H**), C200W30m (**I**) and C200W60m (**J**).

The DSC curves of all collagen fibril gels (Figure 5A) showed a typical thermal denaturation peak induced by heating. DSC spectra showed that the melting temperature (Tm) of collagen fibril gels increased from 50.5 °C (C0W0m) to 52.1 °C (C200W10m) with the ultrasonic treatment power increased from 0 W to 200 W but decreased to 47.8 °C at 400 W (C400W10m); Tm decreased from 53.5 °C (C200W5m) to 46.3 °C (C200W60m) with the ultrasonic treatment time increased from 5 min to 60 min. The enthalpy (ΔH) of collagen fibril gels decreased from 3.19 J/g (C0W0m) to 1.39 J/g (C400W10m) with the ultrasonic treatment power increased from 0 W to 400 W (10 min). ΔH values also showed a descending trend from 2.78 J/g (C200W15m) to 1.86 J/g (C200W60m) with the ultrasonic treatment time increased from 5 min to 60 min (200 W). Tm values depended on the nucleation stage of collagen fibrillogenesis process, and moderate ultrasonic treatment accelerated the nucleation stage to increase the fibrillogenesis degree and thereby strengthen the thermal stability [4]. The amount of intermolecular and intramolecular hydrogen bonds of collagen determined ΔH values. Ultrasonic treatment decreased the number of formed hydrogen bonds and thereby induced a lower ΔH values [8]. These results suggested that ultrasonic treatment will enhance the thermal stability and thus greatly expand the applied potency of collagen fibrils and collagen fibril gels, which may be related to the change in collagen conformation induced by ultrasonic treatment.

#### 2.3.2. Thermo-Gravimetric Analysis (TGA)

The further thermal destruction of collagen fibril gels in a large temperature range was characterized by thermogravimetric curves (TGA) and its first derivatives (DTG) (Figure 5B–J) as well as weight loss per 100 °C (Table S6). The three peaks observed in all DTG plots revealed a three-stage thermal destruction of collagen fibril gels (Figure 5B–J). An initial thermal transitional change was observed at 52.94–200.61 °C, which was attributed to the removal of free water [24]. A maximum percentage transitional weight loss of 5.08% at 168.23 °C (C0W0m) to 15.86% at 156.97 °C (C400W10m) and 11.22% at 148.50 °C (C200W5m) to 18.74% at 156.32 °C (C200W60m) demonstrated that ultrasonic treatment accelerated the loss of free water. A greater water loss of ultrasonic treatment collagen suggested that they were more permeable and thereby suitable for using as carrier or catalyst [24]. In the second stage, collagen binding water, and then small molecular thermal degradation products, such as peptides and amino acids, were released [31]. C0W0m (55.17% at 391.52 °C) exhibited a minimum percentage of transitional weight loss and transitional temperature than that of ultrasonic treatment collagen (62.70–75.19% and 433.43–471.69 °C), which may be due to the fewer hydrogen bonds in the collagen induced by ultrasonic treatment. In the third stage, high temperature further decomposed peptides and amino acids into CO_2_, CO and NO via deamination and dehydration [31], and all the collagen fibril gels completely decomposed. These results suggested that moderate ultrasonic treatment enhanced the impermeability of collagen fibril gels, but high power and/or longtime ultrasonic treatment (*p* > 200 W, t > 15 min) may weaken the thermal stability of collagen fibril gels in a large temperature range.

### 2.4. Viscoelasticity of Collagen Fibril Gels (Dynamic Frequency Sweep Test)

**Figure 6:**
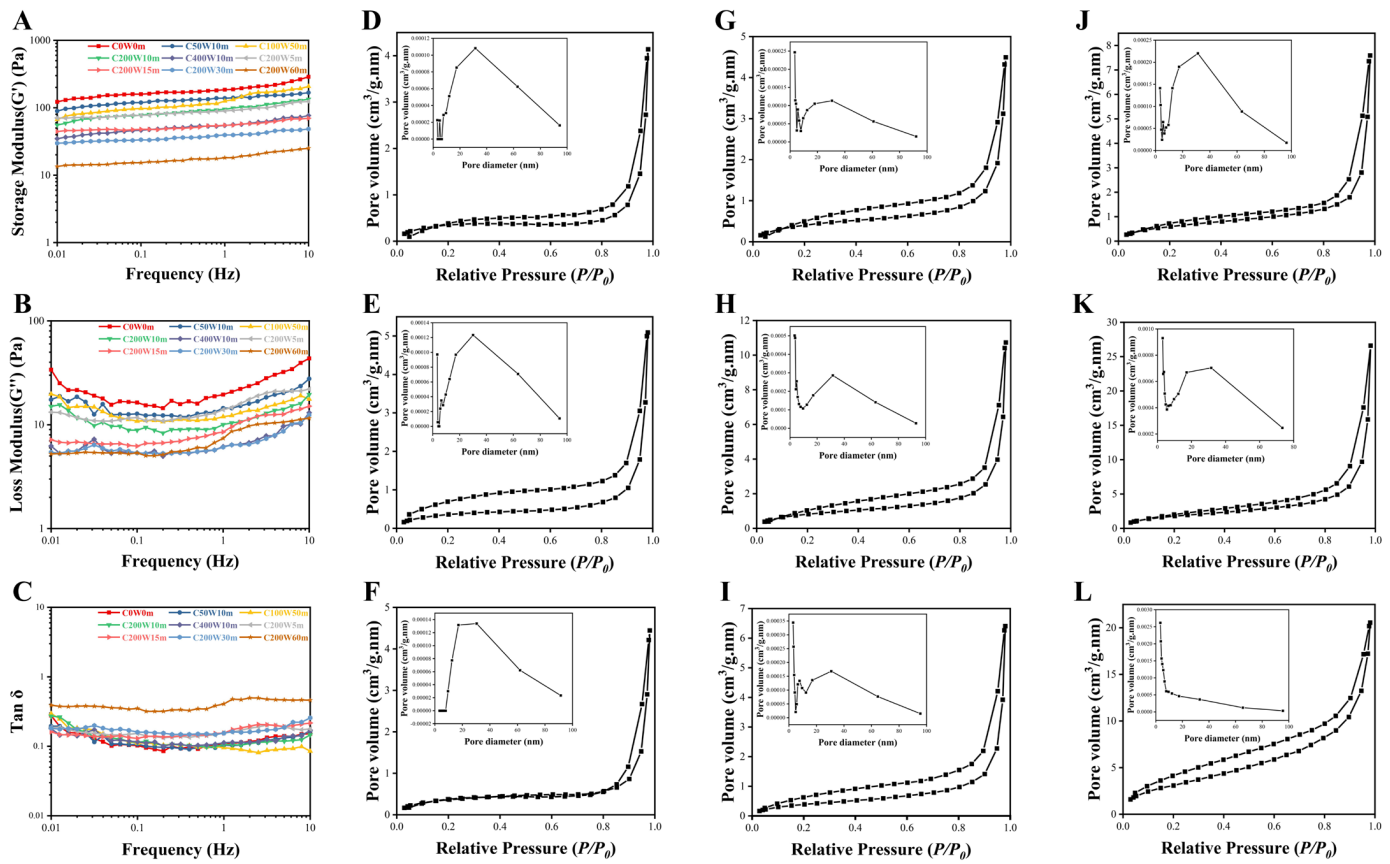
Storage modulus (G′), (**A**), loss modulus (G′′), (**B**) and loss tangent (tan δ), (**C**) of collagen fibril gels; N_2_ adsorption isotherms and BJH pore size distribution curves of C0W0m (**D**), C50W10m (**E**), C100W10m (**F**), C200W10m (**G**), C400W10m (**H**), C200W5m (**I**), C200W15m (**J**), C200W30m (**K**) and C200W60m (**L**).

The viscoelastic properties of collagen fibril gels were evaluated by storage modulus (G′, describes the elasticity behavior) and the loss modulus (G′′, describes the dissipated energy as a characteristic viscosity) obtained from dynamic frequency sweep tests [32,33]. G’ represents the structure stiffness to deform with the impact of external force; it depends on the number and strength of the secondary bonds, while G′′ modulus reflects the energy loss induced by collagen intra- or inter-molecular stretching when the external force changes [9]. As was shown in Figure 6A,B, the G′ and G′′ moduli of all gels increased when the shear frequency increased from 0.01 to 10 Hz, while the G′ modulus was much larger than the corresponding G′′, which was the typical rheological attribute of the gel structure. The G′ and G′′ moduli of ultrasonic treatment collagen generally decreased with increased ultrasonic treatment power and time than that of C0W0m, indicating that ultrasonic treatment could have impaired the viscoelasticity of collagen fibril gels during self-assembly [34,35].

Ultrasonic treatment may alter the entanglement networks between collagen macromolecules as well as configurational rearrangements by short-range relaxation times (between entanglements) and long-range relaxation times (beyond entanglements), thereby endowing the reduced dynamic viscoelastic behavior of collagen fibril gels [35]. Tan δ (G′′/G′, defined as tangent loss angle) represented the mechanical loss of polymer in oscillatory motion, reflecting the network structure of gel. The smaller the tan δ value, especially when tan δ < 0.3, the better the gel network structure formed by the protein [36,37]. It was found that all the ultrasonic treatment collagen showed a smaller tan δ value (tan δ < 0.3, Figure 6C) than that of C0W0m, except for C200W60m (tan δ > 0.3), suggesting that ultrasonic treatment was conducive to the formation of collagen fibril gel network.

### 2.5. Gel Strength

Gel strength represents the transition capacity of colloidal sol into polymer gel. As was shown in Figure 1F, a gradual descending trend of collagen fibril gel strength along with increases in ultrasonic treatment power and time were observed. Ultrasonic treatment during the nucleation stage of collagen fibrillogenesis endowed collagen molecules a higher stretching degree, and the increased hydrophobicity weakened the collagen–water interactions. Therefore, ultrasonic treatment created more tenuous and looser collagen fibril gels compared with C0W0m, and then the collagen fibrillar gel strength was diminished to be “softer” gels. Collagen gels are widely used as biomedical materials in the fields of scaffolds, delivery matrices, catalysts, and injectable carriers. Wherein the biomedical scaffolds require sufficient strength and elasticity, the soft and low-viscosity are more suitable for delivery matrices [7]. In this study, ultrasonic treatment affected the nucleation stage of the collagen fibrillogenesis process, and then reduced the collagen fibril gel strength, which greatly expanded the applied potency of collagen fibril gel.

### 2.6. Nitrogen Adsorption of Collagen Fibril Gels

The nitrogen adsorption isotherm and pore size distribution of collagen fibril gels were shown in Figure 6D–L, and the hysteresis loops suggested they were all type IV isotherms [18]. The specific surface area of collagen fibril gels calculated by the BET method increased from 1.380 m^2^/g to 3.179 m^2^/g when ultrasonic treatment power was 0~400 W (10 min), and increased as well from 1.529 to 12.013 m^2^/g when ultrasonic treatment time was 5~60 min (200 W). BJH analysis (Table S7) showed that the total pore volumes of collagen fibril gels generally showed an increased trend from 0.0064 cm^3^/g to 0.0166 cm^3^/g, and their average pore diameter decreased from 22.466 nm to 16.916 nm when the ultrasonic treatment power was 0~400 W (10 min). The total pore volumes and average pore diameter of collagen fibril gels increased from 0.0099 cm^3^/g to 0.0318 cm^3^/g and decreased from 25.917 nm to 10.581 nm, respectively, when the ultrasonic treatment time was 5~60 min (200 W). BJH analysis showed that high power and longtime ultrasonic treatment created mesoporous collagen fibril gels with larger specific surface area and pore volume but more homogeneous pore diameter than natural fibrils (specific surface area 1~5 m^2^/g). Consequently, these results indicated that ultrasonic treatment may be a potential method to manufacture homogeneous porous collagen scaffolds and biomaterials as catalyst and carrier.

## 3. Materials and Methods

### 3.1. Raw Materials and Chemical Reagents

Freeze-dried bovine bone collagen was prepared by our previous study, which was characterized as type I collagen with the molecular form of [α1(I)]2α2(I) [14]. All other reagents and chemicals used in this study were of analytical grade and obtained from Sinopharm Chemical Reagent Co., Ltd. (Shanghai, China).

### 3.2. Ultrasonic Treatment on Collagen

Bovine bone collagen I was dialyzed in a phosphate buffer (200 mM, pH 7.4) at 4 °C for 48 h after being stirred to dissolve in 10 mM HCl solution (pH 2.0) at a concentration of 5 mg/mL. Subsequently, 200 mL of collagen solutions was incubated with different power and duration of ultrasonication in a glass beaker using an ultrasonic processor (VCX 750, Sonics & Materials Inc., Newtown, CT, USA) equipped with a high-grade titanium alloy probe. The output power was adjustable (0–100%) with a rated power of 750 W (20 kHz) and emitting surface of 13.0 mm diameter. The temperature of the glass was maintained at 30 °C by a temperature-controlled steel jacket through a circulating water bath (30 °C), and the ultrasonic processor worked in a pulse mode of 2 s acting and 3 s resting time to avoid overheating [9]. The probe was immersed in the solution to a depth of 3 cm from the bottom during the ultrasonic treatment process. The collagen solutions were first treated with different ultrasonic treatment power (0–400 W) for 10 min to investigate the effect of ultrasonic treatment power on collagen self-assembly behavior; then, the optimal ultrasonic treatment power (200 W, according to fibrillogenesis rate and fibrillogenesis degree) was selected to study the effect of ultrasonic treatment time (0–60 min) on the collagen self-assembly behavior. The ultrasonic treatment collagen was termed as C0W0m, C50W10m, C100W10m, C200W10m, C400W10m, C200W5m, C200W15m, C200W30m, and C200W60m, respectively (Table 1). After the ultrasonic treatment process, all the collagen solutions (200 mL) were further incubated at 30 °C to 60 min, and 100 mL of the collagen solutions were freeze-dried for further utilization. The actual output ultrasonic treatment power (W) was calculated using the following Equation (1), and the ultrasonic treatment intensity (W/cm^2^, Table 1) equals to the output power divided by the emitting surface (1.327 cm^2^) [38].

(1) $$P\;=\;m\times C_{p}\times \partial T/\partial t$$

where P is the power output, m is the mass of the sonicated liquid (g), C_p_ is the specific calorific capacity of the fluid (J/g °C), and ∂T/∂t is the temperature change rate of the solvent over a 3 min period. In this experiment, the ultrasonication intensity was 0.62–38.47 W/cm^2^ calculated by a calorimetric study of ultrasonic processor VCX 750 (Table 1).

### 3.3. Turbidity Assay

Fibrillogenesis process of bone collagen I was monitored by the turbidity assay according to Tian et al. (2021) [4], because the turbidity of collagen solutions changes during fibrillogenesis in simulated body fluid solution (SBF). The obtained ultrasonic treatment collagen solutions in Section 3.2 (4 mL) were mixed with 1 mL of 10× *g* concentrated SBF (Table S1) at 4 °C, and the solutions were adjusted to pH 7.4 with 2 M NaOH to prepare the collagen stock solutions. The collagen stock solutions were transferred into a quartz cuvette (1 cm) and then incubated in a spectrophotometer (PE Lambda 25, Perkin Elmer, Waltham, MA, USA) at 30 °C for 60 min. The absorbance of collagen mixtures at 313 nm was recorded every 35 s by a UV spectrophotometer (PE Lambda 25, Perkin Elmer, Waltham, MA, USA) during fibrillogenesis.

### 3.4. Fibrillogenesis Degree

Fibrillogenesis degree of collagen is defined as the percent of collagen molecules that reassemble into the fibrils [18]. The fibrillogenesis process was conducted as described in Section 3.3 at 30 °C for 12 h, then the collagen mixtures were centrifuged at 18,000× g (20 °C) for 20 min. The protein content in supernatant was measured by a Lowry protein quantification assay kit, and the collagen fibrillogenesis degree was calculated using Equation (2):

(2) $$Y\left(\%\right)=\frac{C_{0}-C}{C_{0}}\times 100\%$$

where Y is the collagen fibrillogenesis degree (%), C_0_ (mg/mL) and C (mg/mL) is the protein concentration before and after collagen fibrillogenesis.

### 3.5. Microstructure of Ultrasonic Treatment Collagen

#### 3.5.1. Sodium Dodecyl Sulphate Polyacrylamide Gel Electrophoresis (SDS-PAGE)

SDS-PAGE was performed according to the method of Thuy et al. (2014) [39] with slight modifications using BIO-RAD Mini-PROTEAN gels system (Bio-Rad Laboratories, Hercules, CA, USA). The lyophilized collagen was dissolved in 0.1 M acetic acid into a concentration of 5 mg/mL. Then, collagen solutions were mixed with sample buffer (0.5 M Tris–HCl, pH 6.8, containing 10% (*w*/*v*) SDS, 20% (*v*/*v*) glycerol and 0.02% bromophenol blue) at a ratio of 1:1 (*v*/*v*). Each sample (approximately 10 µg) was loaded onto the gels and the electrophoresis was conducted on 80 V for 4% stacking gel and 110 V for 7.5% resolving gel. Gels were stained with 0.1% (*v*/*v*) Coomassie brilliant blue R250 and then decolorized by the decolorizing solution (30% methanol + 10% acetic acid, *v*/*v*). Gels were preserved in 7% (*v*/*v*) acetic acid until they were photographed by the Alpha Ease FC gel imaging system (Alpha Innotech, San Leandro, CA, USA). Quantitative analysis of collagen band intensity was performed using ImageJ 1.8.0 software (National Institute of Mental Health, Bethesda, MD, USA).

#### 3.5.2. Fourier Transform Infrared Spectroscopy (FT-IR)

FTIR spectra of lyophilized collagen were recorded in the range of 4000–400 cm^−1^ with 1 cm^−1^ resolution for a single scan using a Nicolet iS10 FT-IR spectrometer (Thermo Fisher Scientific Inc., Madison, WI, USA) according to the procedure depicted by Kang D et al. (2016) [40]. FT-IR spectra data were analyzed with OMNIC software (v8.20, Thermo Nicolet, Madison, WI, USA).

#### 3.5.3. X-ray Diffraction (XRD)

XRD spectra of lyophilized collagen were obtained following the method described by Pezeshk S et al. (2022) [41] with slight modification using an XRD instrument (XRD-6000, Shimadzu, Kyoto, Japan) with copper Kα as a source of X-rays: scanning range 5–60° (2θ); scanning speed 2°/min. The minimum value of the repeated spacings (d values) was calculated by the Bragg equation, as in Equation (3):

(3) $$d\left(Å\right)=\;\lambda /2\;\sin\theta$$

where λ is the wavelength of copper Kα X-ray (1.54 Å) and θ is the Bragg diffraction angle. 

#### 3.5.4. Fluorescence Emission Spectra

Endogenous fluorescence is produced by aromatic amino acids (tryptophan, tyrosine, phenylalanine) for their benzene ring or conjugated double bond structure [42]. The content of tryptophan and phenylalanine are low in collagen, and thus, tyrosine was selected as the endogenous fluorescence of collagen to analyze the effect of ultrasonic treatment on collagen conformation. According to the method described by Soumya N. et al. (2021) [15], ultrasonic treatment collagen solutions in Section 3.2 were diluted with 10 mM HCl to a concentration of 0.5 mg/mL. The fluorescence spectrum of collagen solutions was recorded using a fluorescence spectrophotometer (F-4600, Hitachi, Tokyo, Japan). The excitation wavelength was 280 nm, the emission wavelength 300–400 nm, the excitation and emission spectral slit 5 nm, while the scanning rate was 1200 nm/min.

### 3.6. Microscopic Structure of Collagen Fibrils (SEM/TEM)

The morphology of collagen fibrils was observed using scanning electron microscope (SEM; S4800, Hitachi, Tokyo, Japan) according to the procedures described by Ran et al. (2020) [16]. Collagen stock solutions (1 mg/mL) were prepared as described in Section 3.2, and 50 μL of collagen stock solutions were incubated overnight at 30 °C in clean slides. The treated samples were rinsed carefully with deionized water for several times and fixed with 2.5% (*v*/*v*) glutaraldehyde for 2 h, then dehydrated with 50% (*v*/*v*) ethanol and freeze-drying with a freeze dryer (SCIENTZ-10ND, Ningbo Xinzhi Biotechnology Co. ltd., Ningbo, China). The dried samples were observed by SEM at an accelerating voltage of 5 kV, and the diameters of 100 fibrils were analyzed with ImageJ software (v1.51, National Institutes of Health, Bethesda, MD, USA).

Observation of the collagen fibrils by transmission electron microscopy (TEM) were performed according to the method of Liu et al. (2014) [29] with slight modifications. Collagen stock solutions were prepared the same as Section 3.2. One drop of the solution was loaded on a copper grid with 200 mesh size and stained with 1% (*w*/*v*) phosphotungstic acid, and then then washed with deionized water and air dried. The collagen fibrils were observed using a JEM-2100 electron microscopy (JEOL Ltd., Tokyo, Japan) with an accelerating voltage of 200 kV.

### 3.7. Preparation of Collagen Fibril Gels

The ultrasonic treatment collagen solutions (40 mL) were mixed with 10 mL of 10× *g* concentrated SBF at 4 °C, and the solutions were adjusted to pH 7.4 with 2 M NaOH. The collagen mixture solutions were incubated for 12 h at 25 °C to prepare collagen fibril gels, which were then incubated for 12 h and kept at 4 °C for further analyses [4].

### 3.8. Thermal Stability of Collagen Fibril Gels

#### 3.8.1. Differential Scanning Calorimetry (DSC)

The melting temperature (Tm) of collagen fibril gels were quantified using Q2000 Series DSC (TA Instruments, Inc., New Castle, DE, USA). Samples (approximately 5 mg) were accurately weighted and sealed into the aluminum pan, and an empty aluminum pan was used as the reference. The pan was equilibrated at a rate of 5 °C/min and heated from 10 to 100 °C [9].

#### 3.8.2. Thermogravimetric Analysis (TGA)

The thermal properties of the collagen fibril gels were evaluated using a TG/DTA instrument (Pyris Diamond 6000 TG/DTA, PerkinElmer, USA) from 40–700 °C at 10 °C/min in a nitrogen atmosphere, and the reported data were averages of three scans [43].

### 3.9. Collagen Fibril Gel Strength

The strength of collagen fibril gels (height 30 mm × diameter 20 mm) was measured following the method of Jiang et al. (2016) [7] using a TA-XT2i texture analyzer (Stable Micro Systems, Surrey, UK). The strength of collagen fibril gels was determined using a cylindrical probe (P/0.5) at a constant velocity of 1.0 mm/s, with five samples for each determination.

### 3.10. Viscoelasticity Properties of Collagen Fibril Gels

The dynamic viscoelasticity of collagen fibril gels was measured following the procedure of Tian et al. (2021) [4] through dynamic frequency sweep tests from 0.01 to 10 Hz at 25 °C using a rheometer (MCR 302, Anton Paar, Austria). The rheometer was equipped with a temperature-controlled stainless-steel cone/plate geometry (0.5° cone angle, 60 mm cone diameter, 57 μm gap). Storage modulus (G′), loss modulus (G′′), and loss tangent (tan δ) were recorded.

### 3.11. Nitrogen Adsorption (BET) of Collagen Fibril Gels

The specific surface areas, pore size and pore volume of collagen fibril gels were determined by a surface area analyzer (Autosorb-6B, Quantachrome Instruments, Boynton Beach, FL, USA) based on the Brunauer–Emmett–Teller (BET) method and the Barrett–Joyner–Halenda (BJH) method, following the procedure described by Liao et al. (2018) [18]. The pore structure information of collagen fibril gels was calculated from the desorption branch.

### 3.12. Statistical Analysis

All experiments were made in triplicate and results were presented as mean ± standard deviation (SD). Statistical analyses were analyzed using ANOVA’s test (*p* < 0.05) by SPSS 26.0 (IBM SPSS Statistics, Ehningen, Germany) and all the figures were processed by Origin 9.0 software (OriginLab Co., Northampton, MA, USA).

## 4. Conclusions

Results of SDS-PAGE, FT-IR, XRD and fluorescence emission spectra showed that ultrasonic treatment did not damage the integrity of collagen triple-helical structure, but induced multi-hierarchical structural alteration and dispersed the collagen aggregates evenly. Proper ultrasonic treatment (*p* ≤ 200 W, t ≤ 15 min) accelerated collagen self-assembly rates and gained a decreased but more homogeneous fibril diameter and D-periodicity lengths. Proper ultrasonic treatment (*p* ≤ 200 W, t ≤ 15 min) also impaired the viscoelasticity properties and enhanced the thermal stability of collagen fibril gels, promoting specific surface area and porosity of collagen fibril gels while creating a softer gel. Consequently, this study may contribute a new avenue for controlling collagen fibrillogenesis in vitro and ameliorating collagen fibril gel properties to manufacture novel collagen biomaterials with desirable performances for further valorization. However, the optimum ultrasonic treatment power and duration should be carefully evaluated depending on the requirement.

## Figures and Tables

**Table 1:** Calorimetric study on VCX 750 ultrasonic probe and ultrasonication conditions for collagen samples.

Samples	Power Setting (W)	Actual Power Output (W)	Ultrasonic Time at 30 °C (min)	Ultrasonication Intensity (W/cm^2^) ^a^	Incubation Time at 30 °C in the Absence of Ultrasonic (min)
C0W0m	0	0	0	0	60
C50W10m	50	0.82	10	0.62	50
C100W10m	100	1.56	10	1.15	50
C200W10m	200	8.32	10	6.27	50
C400W10m	400	51.05	10	38.47	50
C200W5m	200	8.32	5	6.27	55
C200W15m	200	8.32	15	6.27	45
C200W30m	200	8.32	30	6.27	30
C200W60m	200	8.32	60	6.27	0

^a^: Ultrasonic intensity is equal to the power output measured by calorimetry divided by the area of the emitting surface (1.327 cm^2^).

## Data Availability

Not applicable.

## Supplementary Materials

The following supporting information can be downloaded at: https://www.mdpi.com/article/10.3390/molecules28073096/s1, Table S1: Components of simulated body fluid solution (mM); Table S2: SDS-PAGE band intensities of collagen; Table S3: Fourier transform-infrared spectra peak wavenumber and assignment of collagen; Table S4: Curve-fitting analysis of amide I band of collagen; Table S5: D values calculated by the Bragg equation of the two x-ray diffraction peaks of collagen; Table S56: Thermal analysis of collagen fibril gels under N_2_ air atmosphere; Table S7: N_2_ adsorption analysis of collagen fibril gels.
