# Supplementary material for: Impact of Ultrasonication on the Self-Assembly Behavior and Gel Properties of Bovine Bone Collagen I
Source: Molecules. 2023 Mar 30;28(7):3096. doi: 10.3390/molecules28073096 (PMC10095610; doi:10.3390/molecules28073096)
Supplement: Supplementary file 1 [file molecules-28-03096-s001.zip › molecules-2164231-supplementary.pdf]

Supplementary Files **(Color should be used for all figures)**

Supplementary file S1.
Table S1 Components of simulated body fluid solution (mM).

Supplementary file S2.
Table S2 SDS-PAGE band intensities of collagen.

Supplementary file S3.
Table S3 Fourier transform-infrared spectra peak wavenumber and assignment of collagen.

Supplementary file S4.
Table S4 Curve-fitting analysis of amide I band of collagen

Supplementary file S5.
Table S5 D values calculated by the Bragg equation of the two x-ray diffraction peaks of collagen.

Supplementary file S6.
Table S6 Thermal analysis of collagen fibril gels under N_2 air atmosphere.

Supplementary file S7.
Table S7 N_2 adsorption analysis of collagen fibril gels.

Supplementary material S1

Table S1 Components of simulated body fluid solution (mM).

NaCl	$NaHCO_3$	KCl	$K_2HPO_4 \cdot 3H_2O$	$CaCl_2$	Na_2SO_4	$MgCl_2 \cdot 6H_2O$	Tris
1 370	42	30	10	25	5	15	300

Supplementary material S2

Table S2 SDS-PAGE band intensities of collagen

Samples	Bands	Band intensities						
		Area	Mean gray value	Standard deviation	Min gray value	Max gray value	Raw density	α1/α2 Ratio
C0W0m	Lane	-	-	-	-	-	5584852	2.032
	γ-band	7446	109.912	6.591	90	135	818463	
	β-band	17920	94.155	7.222	80	123	1687258	
	α1-band	23808	86.676	17.487	61	138	2063586	
	α2-band	10096	100.591	8.893	83	131	1015545	
C50W10m	Lane	-	-	-	-	-	5063284	2.037
	γ-band	7632	106.787	5.164	91	127	815002	
	β-band	15232	91.593	5.878	80	121	1395139	
	α1-band	23217	82.428	16.085	59	126	1913682	
	α2-band	9225	101.851	8.672	85	129	939461	
C100W10m	Lane	-	-	-	-	-	4598648	2.055
	γ-band	8272	97.462	6.535	85	126	806208	
	β-band	13600	90.896	6.523	75	116	1236182	
	α1-band	20064	85.701	17.172	61	127	1719512	
	α2-band	8348	99.168	10.446	82	130	836746	
C200W10m	Lane	-	-	-	-	-	6113291	2.021
	γ-band	6476	116.124	7.306	94	139	751948	
	β-band	17136	93.566	7.390	80	119	1603342	
	α1-band	28400	88.508	18.926	61	134	2513630	
	α2-band	11576	107.492	8.890	85	136	1244371	
C400W10m	Lane	-	-	-	-	-	5102817	1.963
	γ-band	5088	106.761	6.941	89	123	543198	
	β-band	14336	98.796	7.678	80	119	1416341	
	α1-band	23552	88.419	17.799	61	131	2082435	
	α2-band	9904	107.115	9.393	81	136	1060843	
C200W5m	Lane	-	-	-	-	-	5052256	2.019
	γ-band	8001	106.928	6.616	90	137	855427	
	β-band	13872	92.667	6.608	80	112	1285483	
	α1-band	24165	80.573	16.321	56	132	1947005	
	α2-band	8956	107.687	8.777	85	136	964341	
C200W15m	Lane	-	-	-	-	-	4769901	2.028
	γ-band	6528	98.923	5.156	84	113	645770	
	β-band	15232	92.767	6.658	82	118	1413032	
	α1-band	22943	79.142	16.528	55	121	1815756	
	α2-band	8781	101.969	10.054	83	126	895343	
C200W30m	Lane	-	-	-	-	-	4745278	2.006
	γ-band	6022	100.980	8.352	87	134	608050	
	β-band	11089	91.693	7.187	70	116	1016727	
	α1-band	24192	86.078	15.557	61	125	2082410	

	α2-band	10400	99.819	7.957	84	121	1038091	
	Lane	-	-	-	-	-	4770263	
	γ-band	5358	109.396	15.584	81	182	586131	
C200W60m	β-band	9803	102.820	9.649	80	140	1007875	1.983
	α1-band	23232	90.886	17.466	65	161	2111471	
	α2-band	10013	106.336	19.079	79	167	1064786	

Lane, the overall intensities of γ-band, β-band, α1-band, and α2-band.

Supplementary material S3

Table S3 Fourier transform-infrared spectra peak wavenumber and assignment of collagen

Region	Wavenumber (cm^{-1})									Assignment
	1	2	3	4	5	6	7	8	9	
Amide A	3298	3292	3292	3294	3298	3294	3307	3325	3325	N-H stretch coupled with hydrogen bonding
Amide B	2922	2924	2926	2934	2928	2924	2926	2932	2932	CH_3-asymmetric stretch
	2853	2853	2855	2880	2874	2852	2876	2876	2876	CH_2 symmetric and asymmetric stretch
Amide I	1661	1661	1653	1651	1655	1651	1649	1649	1634	C=O stretch/hydrogen bond coupled with COO^-
Amide II	1518	1531	1547	1553	1541	1533	1551	1520	1535	N-H bend coupled with C-N stretch
	1454	1454	1452	1454	1452	1452	1452	1449	1449	CH_2 bend
	1389	1389	1402	1404	1394	1391	1402	1389	1387	COO^- symmetric stretch
	1344	1342	1337	1339	/	1344	1338	1342	1342	CH_2 wag
Amide III	1230	1234	1238	1240	1240	1234	1238	1230	1236	N-H bend coupled with C-N stretch
	1173	1173	1165	1163	1171	1175	1161	1159	1159	C–O stretch/C–N–C stretch
	1078	1076	1082	1082	/	1072	1080	1070	1069	Skeletal stretch
$A_{III}/A_{1\,450}$	1.1299	1.0841	1.1044	1.0575	1.0776	1.1301	1.0487	1.0855	1.0880	Integrity of triple helix structure

/: no peak. 1: C0W0m; 2: C50W10m; 3: C100W10m; 4: C200W10m; 5: C400W10m; 6: C200W5m; 7: C200W15m; 8: C200W30m; 2: C200W60m

Supplementary material S4

Table S4 Curve-fitting analysis of amide I band of collagen

Secondary structure	Ultrasonic/non-ultrasonic treatment collagen								
	C0W0m	C50W10m	C100W10m	C200W10m	C400W10m	C200W5m	C200W15m	C200W30m	C200W60m
From fitting by Gaussian equation									
α-helices	58.35%	44.26%	36.75%	35.40%	42.58%	36.81%	20.50%	18.22%	16.11%
β-sheets	34.92%	42.59%	36.82%	29.95%	23.06%	31.15%	39.99%	38.74%	42.55%
β-turns	6.73%	13.13%	26.32%	31.26%	18.66%	22.10%	29.81%	33.18%	23.62%
random coils	0.00%	0.02%	0.11%	3.39%	15.70%	9.94%	9.71%	9.86%	17.72%

Supplementary material S5

Table S5 D values calculated by the Bragg equation of the two x-ray diffraction peaks of collagen

Diffraction peaks	Ultrasonic/non-ultrasonic treatment collagen								
	C0W0m	C50W10m	C100W10m	C200W10m	C400W10m	C200W5m	C200W15m	C200W30m	C200W60m
Diffraction angle 1 (°)	8.12	7.98	7.92	7.70	7.66	8.06	7.96	7.56	7.52
d1 (nm)	1.088	1.107	1.115	1.147	1.152	1.096	1.109	1.168	1.174
Diffraction angle 2 (°)	20.86	19.94	19.76	19.66	20.84	20.10	19.90	19.62	20.32
d2 (nm)	0.425	0.445	0.449	0.451	0.426	0.441	0.446	0.452	0.437

Supplementary material S6

Table S6 Thermal analysis of collagen fibril gels under N_2 air atmosphere

Temperature (℃)	Ultrasonic/non-ultrasonic treatment collagen								
	C0W0m	C50W10m	C100W10m	C200W10m	C400W10m	C200W5m	C200W15m	C200W30m	C200W60m
	Weight loss (%)								
100	11.45	11.59	7.61	10.28	4.84	11.44	13.67	10.11	14.05
200	13.17	13.61	10.63	18.16	6.26	13.67	16.45	11.96	16.18
300	32.21	30.01	25.41	20.66	25.51	30.68	34.63	30.46	32.20
400	61.55	61.11	60.04	56.95	56.54	61.01	69.63	58.98	65.13
500	74.02	70.83	67.48	88.83	69.14	70.88	77.34	70.69	72.73
600	94.35	90.13	82.13	96.22	87.81	90.09	90.13	97.74	81.05
700	99.99	99.99	99.99	99.99	99.73	99.99	99.99	99.99	96.45

Supplementary material S7

Table S7 N_2 adsorption analysis of collagen fibril gels

	C0W0m	C50W10m	C100W10m	C200W10m	C400W10m	C200W5m	C200W15m	C200W30m	C200W60m
BET surface area (m^2/g)	1.380	1.401	1.417	1.644	3.179	1.529	2.381	6.801	12.013
Total pore volume (cm^3/g)	0.0064	0.0079	0.0069	0.0069	0.0166	0.0099	0.0117	0.0411	0.0318
Average pore diameter (nm)	18.552	22.466	19.407	16.916	20.878	25.917	19.724	24.194	10.581
